# Impact of Dietary Coparenting and Parenting Strategies on Picky Eating Behaviors in Young Children

**DOI:** 10.3390/nu16060898

**Published:** 2024-03-20

**Authors:** Jo-Lin Chen, Jia-Yau Doong, Miao-Ju Tu, Shou-Chi Huang

**Affiliations:** 1 Child and Family Studies, Fu Jen Catholic University, New Taipei City 242062, Taiwan; 046286@mail.fju.edu.tw (J.-L.C.); 081429@mail.fju.edu.tw (M.-J.T.); 2 Nutritional Science Studies, Fu Jen Catholic University, New Taipei City 242062, Taiwan; 141600@mail.fju.edu.tw

**Keywords:** coparenting, dietary behavior, parenting strategy, picky eating, young child

## Abstract

Many studies have demonstrated that coparenting and parenting behaviors have a substantial effect on the behaviors of young children. Research has indicated that young children may exhibit picky eating behaviors, which pose challenges for parents in terms of coparenting and parenting. This study examined how dietary coparenting and parenting strategies directly affect young children’s picky eating behaviors and explored the mediating role of parenting strategies in the relations between parental dietary coparenting and young children’s picky eating behaviors. More specifically, this study focused on parents of three- to six-year-old children in northern Taiwan. A total of 408 valid completed questionnaires were collected, and the research tools included scales measuring dietary coparenting, parenting strategies, and young children’s picky eating behaviors. The results revealed that supportive and undermining dietary coparenting and parenting strategies had a significant direct effect on young children’s picky eating behaviors. Furthermore, supportive and undermining dietary coparenting partially mediated young children’s picky eating behaviors through parenting strategies. Specifically, among parenting strategies, both “encouraging and facilitating the trying of new foods” and “guiding and modeling” proper eating behaviors had significant indirect effects on reducing young children’s picky eating behaviors.

## 1. Introduction

The preschool period is a crucial time in a person’s life when long-term dietary habits are established. These habits play a pivotal role in shaping the person’s healthy eating patterns later in life [1]. Picky eating is one of the common dietary behaviors in young children [2] and is considered a barrier to healthy eating behaviors [3]. The prevalence of picky eating has been estimated to range from 5.6% to 47%, and this variation in prevalence is due to differences in assessment methods, children’s ages, and the countries in which studies have been conducted [4,5]. A universally accepted definition of picky eating is not yet available, and consensus regarding the most effective tool for identifying it has yet to be reached [6]. Picky eating behaviors include a child’s refusal to eat both familiar and unfamiliar foods, their reluctance to try new foods, and the development of strong preferences for certain foods [7].

Picky eating can lead to a reduction in dietary variety, potentially resulting in an unhealthy diet or one lacking adequate nutrition [6]. Compared with non-picky eaters, picky eaters consume a narrower range of food items [8]. Such limited variety can result in poor growth, being underweight [9,10] or overweight [3], or developing eating disorders [11]. Also, picky eaters may experience constipation due to inadequate intake of dietary fiber, commonly resulting from low fruit and vegetable consumption [12]. The causes of picky eating include early feeding challenges, delayed introduction of textured foods during weaning, external pressure to eat certain foods, and early signs of selective food preferences [13].

Preferences and eating habits established during childhood often persist into adolescence and may even endure into adulthood [14]. In addition, a longitudinal generational study found that children who exhibited picky eating behaviors at 3 years of age tended to continue these behaviors until the ages of 10 and 13 years [15]. Some children who exhibit persistent picky eating tendencies may encounter developmental challenges and may be prone to thinness during adolescence or the development of eating disorders, including picky eating, in adulthood [16]. Another study reported that, compared with children with normal weights, those who were overweight or obese had significantly higher scores for picky eating and food neophobia [3]. Furthermore, a separate study indicated that individuals who were picky eaters as children were more likely to maintain selective eating patterns and emotional undereating in young adulthood, being less likely to consume a nutritious diet in adulthood. These trends highlight the prolonged effect of childhood eating behaviors on one’s diet and body weight, underscoring the importance of early intervention in promoting healthy eating habits [17]. In summary, establishing healthy eating habits in early childhood is crucial.

Picky eating in children can result in a variety of outcomes related to parenting, including parental concerns regarding the child’s growth and health, mealtime conflicts, and parental guilt. Parents often perceive food pickiness as a manifestation of opposition and assertiveness in their children [18]. Thus, picky eating behaviors in young children can cause considerable stress, anxiety, and strain in family relationships [19]. Parents and primary caregivers, being the primary providers of food for young children, play a crucial role in establishing children’s dietary preferences. Strategies and behaviors used by parents and primary caregivers to achieve specific dietary goals for their children are known as food parenting practices [20]. Food behaviors and feeding strategies adopted by parents are the most pivotal factors affecting a child’s eating behaviors and food preferences. Parents serve as key role models by introducing their children to diverse nutritious food choices. Through various feeding practices, parents and primary caregivers can influence the dietary behaviors of young children and guide them toward the achievement of specific dietary objectives [21].

The importance of family mealtimes, routines, and nutritious meals is typically agreed on by couples. However, disagreements can arise during discussions of strategies to restrict unhealthy foods and establish a harmonious atmosphere during family mealtimes [22]. Such disputes are often related to problems such as access to energy-dense, nutrient-poor snacks and the introduction of dietary variety [23].

Parents use effective strategies to address or alleviate picky eating in their children, including repeatedly exposing them to unfamiliar foods, modeling healthy eating behaviors, co-preparing meals, and fostering positive social experiences during mealtimes. However, counterproductive strategies, such as offering rewards for eating, have also been observed. These findings indicate that parents could benefit from higher-quality information regarding the nature of food pickiness and proven strategies for managing it [18]. One study focused on maternal feeding practices, dietary intake, and BMI in 5 year-olds in a multiethnic Asian population. That study determined that higher levels of maternal role modeling, encouragement of a diverse and balanced diet, and instruction regarding nutritional knowledge were associated with higher levels of vegetable and whole grain intake in children [24]. A positive and encouraging approach, such as rewarding children for trying new foods, is more effective in promoting a preference for and acceptance of healthy foods compared with strict or forceful methods [20].

Extensive evidence indicates the crucial roles of both coparenting quality and food-related parenting practices in shaping children’s behavioral outcomes. However, whether coparenting quality affects food-related parenting practices remains unclear [25]. In particular, in Taiwan, few studies have simultaneously explored the relations among dietary coparenting, parenting strategies, and young children’s picky eating behaviors. Accordingly, the findings of this study provide insights into picky eating behaviors and cultural differences among eating behaviors. The present study examined the relations among dietary coparenting, parenting strategies, and children’s picky eating behaviors in children aged 3–6 years in northern Taiwan.

## 2. Materials and Methods

### 2.1. Study Participants

This study focused on the primary caregivers of preschool children aged 3–6 years in northern Taiwan and used a questionnaire survey as the primary research method. Two-parent families with children aged 3–6 were invited to participate, with one parent—who is the primary caregiver for the child’s diet—completing the questionnaire. First, the research team invited and set up an agreement with 14 preschools in northern Taiwan. Next, we conducted briefing sessions during on-site visits to the preschools to explain the study’s content, objectives, and related information to parents. The information, including anonymity, the time to complete the questionnaire, and the incentive, was disclosed, and the researchers addressed any inquiries from parents. The questionnaire included questions regarding the sociodemographic characteristics of the children and parents, dietary coparenting, parenting strategies, and young children’s picky eating behaviors.

After the briefing sessions, the preschools assisted by distributing invitation letters to parents and reported the participant count to the research team based on returned agreement responses. Subsequently, parents who agreed to participate received the questionnaire along with an informed consent form. The questionnaire responses were anonymous to protect the privacy of the participants. The completed questionnaire was returned within 2 weeks in exchange for a small gift. This study distributed 440 questionnaires and received 421 responses, and the return rate was 95.68%. After excluding incomplete responses, 408 valid questionnaires were collected, accounting for 92.73% of the effective rate. This study was approved by the Institutional Review Board of Fu Jen Catholic University in New Taipei City, Taiwan (FJU-IRB NO: C107124). Informed consent was obtained from all the participants involved in this study.

### 2.2. Measurements

#### 2.2.1. Sociodemographic Characteristics

Data were collected regarding each child’s sex and birth month and the age and educational level of both the father and mother. In addition, the parents were requested to report their child’s current weight and height, and BMI scores were also calculated. Each child’s weight status was determined on the basis of the relevant World Health Organization criteria [26].

#### 2.2.2. Dietary Coparenting Scale

The Dietary Coparenting Scale was modified from a previous coparenting scale [27], and content validity was established by experts’ comments. This scale consists of two types of coparenting: supportive and undermining. Each subscale has five questions. Responses are rated on a 5 point Likert scale (1 = strongly disagree; 2 = disagree; 3 = neutral; 4 = agree; and 5 = strongly agree). A higher score denotes more dietary support or undermining. Through exploratory factor analysis, the two subscales explained 78.14% of the variance. The Cronbach’s α scores were 0.93 for supportive coparenting and 0.94 for undermining coparenting. The scale in this study demonstrated high reliability and was validated.

#### 2.2.3. Parenting Strategies Scale

The Parenting Strategies Scale, inspired by the past literature [18,28], was developed through parents’ focus groups, content validity determined by experts, and factor analysis. It encompasses three dimensions: encouraging and facilitating the trying of new foods, copreparing meals and providing meal variations, and guiding and modeling. Each subscale has five questions. Responses are scored on a 5 point Likert scale (1 = never; 2 = seldom; 3 = sometimes; 4 = usually; and 5 = always). The total score of each item is calculated. A higher score indicates greater use of the given parenting strategy. Through exploratory factor analysis, the three subscales explained 52.54% of the variance. The Cronbach’s α scores of encouraging and facilitating the trying of new foods, copreparing meals and providing meal variations, and guiding and modeling subscales were 0.73, 0.70, and 0.72, respectively, indicating that the scale had high internal consistency reliability. The scale in this study was reported to show high reliability and was validated.

#### 2.2.4. Young Children’s Picky Eating Behavior Scale

The Young Children’s Picky Eating Behavior Scale, used to assess children’s picky eating behaviors in this study, was developed and grounded in a definition of fussy eating [2] and underwent expert validation. The scale was then revised on the basis of the “food fussiness” subscale in the Children’s Eating Behavior Questionnaire [29]. The Young Children’s Picky Eating Behavior Scale comprises four questions, each rated on a 5 point Likert scale (1 = never; 2 = seldom; 3 = sometimes; 4 = usually; and 5 = always), with reversed scoring for specific questions. A higher average score indicates more pronounced picky eating behaviors. Through exploratory factor analysis, the total variance was 64.51%. The scale’s Cronbach’s α score was 0.79, indicating that it had high internal consistency reliability. Subsequently, a criterion-related validity analysis was conducted on items related to children’s dislike of various types of food, including vegetables, fruits, meat, seafood, and rice or noodles alone. For five questions, we observed a significant, medium-to-low positive correlation, with the correlation coefficient ranging from 0.33 to 0.66 (*p* < 0.001). This finding confirmed the construct validity of the scale, thereby supporting its reliability and appropriacy for measuring the intended construct.

### 2.3. Statistical Analysis

This study used SPSS Statistics version 25.0 for exploratory factor analysis and Cronbach’s α analysis to select questionnaire items. Descriptive statistics were used to analyze the characteristics of the participants and variables. Pearson correlation was conducted to explore the associations among background variables, dietary coparenting, parenting strategies, and young children’s picky eating behaviors. In addition, mediation analysis was performed using PROCESS [30] to examine the mediating role of parenting strategies in the relations between coparenting and young children’s picky eating behaviors. Where mediating effects were discovered, linear interactions were calculated using PROCESS for the percentiles of the mediator. Because of the nonnormal distributions of some of our variables, bootstrapping with 5000 samples was used to generate 95% confidence intervals (CIs) for the interaction parameter calculated in the mediation analysis. A *p* value < 0.05 indicated statistical significance.

## 3. Results

### 3.1. Participants’ Characteristics

This study assessed 408 parents of young children, namely 348 (85.3%) mothers and 60 (14.7%) fathers. The mothers’ ages ranged from 24 to 48 years, with an average age of 37.49 years. The fathers’ ages ranged from 25 to 57 years, with an average age of 39.66 years. Most of the mothers (66.9%) had completed education to the college level, as had 54.2% of the fathers (Table 1).

Regarding the basic information of the young children, this study assessed 229 (56.1%) boys and 179 (43.9%) girls. The average age of the children was 64.52 months. The heights of the children ranged from 90 to 130 cm, with an average height of 111.40 cm. Their weights ranged from 12 to 34 kg, with an average weight of 19.18 kg. When categorizing the children’s BMI on the basis of their heights and weights relative to their ages, the majority fell into the “normal weight” category (75.0%), whereas 11.0%, 6.9%, and 7.1% were classified as underweight, overweight, and obese, respectively (Table 1).

### 3.2. Dietary Coparenting, Parenting Strategies, and Children’s Picky Eating Behaviors

#### 3.2.1. Dietary Coparenting

Dietary coparenting was divided into two types, namely supportive and undermining. The scores for supportive coparenting (M = 3.83) were higher than those for undermining coparenting (M = 1.82). All the questions related to supportive coparenting tended to be consistent, with “When I ask my spouse for help in disciplining our child’s eating behavior, my spouse is willing to assist” (M = 3.95) achieving the highest score and with “My spouse provides additional explanations to our child regarding my expectations for eating behavior” (M = 3.67) achieving the lowest score. By contrast, all the questions related to the undermining coparenting aspect tended to be inconsistent, with “My spouse criticizes my approach to disciplining our child’s eating behavior in front of the child” (M = 1.90) achieving the highest score and with “When I handle our child’s eating behavior, my spouse undermines me” (M = 1.72) achieving the lowest score (Table 2).

#### 3.2.2. Parenting Strategies Scale

As mentioned, parenting strategies encompassed three dimensions: encouraging and facilitating the trying of new foods, co-preparing meals and providing meal variations, and guiding and modeling. Among these dimensions, encouraging and facilitating the trying of new foods (M = 3.88) achieved the highest score, indicating that the parents frequently used strategies to encourage their children to try new foods. By contrast, co-preparing meals and providing meal variations (M = 3.00) achieved the lowest score, suggesting that the parents used such strategies relatively infrequently. In terms of encouraging and facilitating the trying of new foods, each item ranged from occasional to frequent use, with “After my child tries a new food, I provide positive encouragement and affirmation” (M = 4.23) achieving the highest score and with “I present different types of food attractively on the plate to encourage my child to try them” (M = 3.23) achieving the lowest score. By contrast, most items related to co-preparing meals and providing meal variations tended to be used occasionally, with “I use multiple cooking methods for my child, including boiling, stir-frying, and pan-frying” (M = 3.81) achieving the highest score and with “I work with my child to make food into cute patterns or shapes” (M = 2.28) achieving the lowest score. Guiding and modeling ranged from occasional to often, with “I facilitate joint dining experiences for my child to dine with their siblings or friends, thereby establishing positive learning opportunities” (M = 3.85) achieving the highest score and with “I play games to help my child become familiar with various foods” (M = 2.69) achieving the lowest score (Table 3).

#### 3.2.3. Young Children’s Picky Eating Behaviors and Food Types

As mentioned, the Young Children’s Picky Eating Behavior Scale comprised four items. After reverse-scoring the relevant question, the overall average score (M = 2.48) indicated that the picky eating behaviors of the young children in this study tended to be “seldom”. Specifically, “My child is picky and eats only or refuses to eat specific types of food (excluding religious or allergy factors)” achieved the highest score (M = 2.93), whereas “My child eats only foods prepared in a particular way” (M = 1.76) achieved the lowest score (Table 4).

Using the average score (i.e., “three points (sometimes)”) as the cutoff for picky eating behaviors, the young children in this study were divided into a picky eating group and a non-picky eating group. The analysis revealed that a total of 31.6% of these young children were considered picky eaters. In addition, an independent samples *t* test was conducted to analyze the preferences for different types of food. The results indicated that the children in the picky eating group had significantly stronger likes and dislikes for certain foods than the children in the non-picky eating group, regardless of whether they liked or disliked those foods (Table 5).

To understand the food types avoided by the young children in the picky eating group, the frequencies of the “usually” and “always” responses were calculated. The results revealed that 15.5% of the children did not like to eat vegetables, 7.6% ate only rice or noodles, 7.4% avoided seafood (excluding allergy reasons), 6.4% avoided meat (excluding religious reasons), and 2.9% did not like to eat fruits. These results indicated that a higher percentage of children exhibited a preference for “does not like to eat vegetables”, whereas a lower percentage of children exhibited a preference for “does not like to eat fruits”.

### 3.3. Results of Correlations among Dietary Coparenting, Parenting Strategies, and Young Children’s Picky Eating Behaviors

The present results indicate that supportive coparenting exhibited a significant low-to-moderate positive correlation with all three parenting strategies (*r* = 0.32–0.41, *p* < 0.001) and a significant moderate negative correlation with picky eating behaviors. Among the findings, more supportive coparenting was found to be significantly associated with more frequent parenting strategies and fewer child picky eating behaviors, and vice versa. Undermining coparenting had a significant low negative correlation with encouraging and facilitating the trying of new foods and guiding and modeling (*r* = −0.17, *p* < 0.001; *r* = −0.15, *p* < 0.01). Undermining coparenting and picky eating behaviors exhibited a significant low positive correlation. The results indicated that more undermining coparenting was significantly related to less frequent encouraging and facilitating of the trying of new foods, while more guiding and modeling was significantly related to more child picky eating behaviors, and vice versa. Finally, the three parenting strategies all displayed a significant moderate negative correlation with picky eating behaviors (*r* = −0.35~−0.46, *p* < 0.001) (Table 6). The results suggested that more encouraging and facilitating of the trying of new foods, co-preparing meals and providing meal variations, and guiding and modeling were linked to fewer child picky eating behaviors reported by parents, and vice versa.

### 3.4. Mediation Analysis

Given the significant associations among dietary coparenting, parenting strategies, and picky eating behaviors, we further investigated the potential mediating roles of parenting strategies (Table 7).

**Figure 1:**
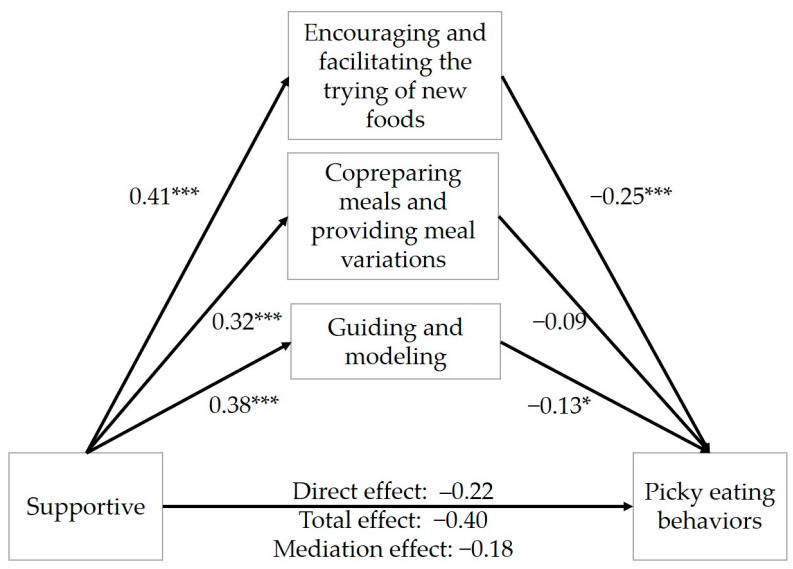
Mediation effects of parenting strategies on the associations between supportive coparenting and picky eating behaviors. * *p* < 0.05. *** *p* < 0.001.

As depicted in Figure 1, with supportive coparenting exerting a negative total effect on picky eating behaviors (β = −0.40, 95% CI: from −0.49 to −0.31), the three parenting strategies partially mediated this association (indirect effect: −0.18, Boot 95% CI: from −0.23 to −0.12; direct effect: β = −0.22, 95% CI: from −0.32 to −0.13), accounting for 45.0% of the total effect (Table 7). Using PROCESS analysis, it was found that encouraging and facilitating the trying of new foods (indirect effect = −0.10, Boot 95% CI: from −0.15 to −0.05) and guiding and modeling (indirect effect = −0.05, Boot 95% CI: from −0.095 to −0.01) both had a significant negative indirect effect on the association between supportive coparenting and picky eating behaviors, and encouraging and facilitating the trying of new foods had the highest effect. However, co-preparing meals and providing meal variations did not have a significant indirect effect on the association between supportive coparenting and picky eating behaviors (indirect effect = −0.03, Boot 95% CI: from −0.07 to 0.01). According to the results, the negative relation between supportive coparenting and picky eating behaviors was significantly mediated by two parenting strategies, encouraging and facilitating the trying of new foods, and guiding and modeling.

**Figure 2:**
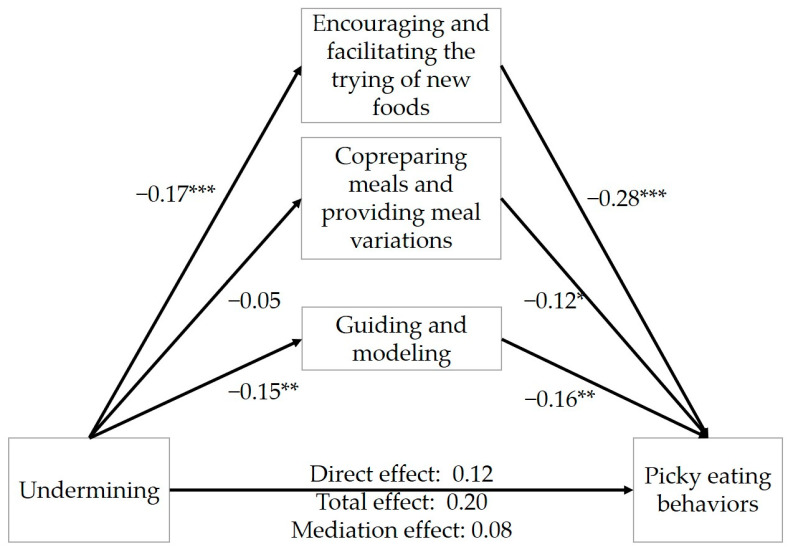
Mediation effects of parenting strategies on the associations between undermining coparenting and picky eating behaviors. * *p* < 0.05. ** *p* < 0.01. *** *p* < 0.001.

As presented in Figure 2, with undermining coparenting exerting a positive total effect on picky eating behavior (β = 0.20, 95% CI: 0.10–0.30), the three parenting strategies partially mediated this association (indirect effect: 0.08, Boot 95% CI: 0.03–0.13; direct effect: β = 0.12, 95% CI: 0.03–0.21), accounting for 40.0% of the total effect (Table 7). It was found that encouraging and facilitating the trying of new foods (indirect effect: 0.05, Boot 95% CI: 0.02–0.09) and guiding and modeling (indirect effect: 0.02, Boot 95% CI: 0.004–0.05) both had a significant, positive indirect effect on the relation of undermining coparenting and picky eating behaviors, and encouraging and facilitating the trying of new foods had the highest effect. Co-preparing meals and providing meal variations did not have a significant indirect effect on the association between undermining coparenting and picky eating behaviors (indirect effect: 0.01, Boot 95% CI: from −0.01 to 0.03). The results indicated that undermining coparenting was significantly positively related to picky eating behaviors through encouraging and facilitating the trying of new foods and guiding and modeling.

## 4. Discussion

In this study, similar to some previous studies [31,32], we classified children with an average score of three (indicating “sometimes”) or higher as picky eaters, where the average score refers to the assessment of young children’s preferences for different types of food. Based on this criterion, we calculated the proportion of children exhibiting picky eating behaviors. We observed that 31.6% of the young children in our study exhibited picky eating behaviors. This proportion was slightly lower than those reported in previous studies [33,34,35]. For example, one study indicated that 42.2% of young children aged between 1 and 5 years were picky eaters [33]. A study conducted in Taiwan reported a prevalence of 54% for picky eating among young children [35]. Variations in the definitions of picky eating and differences in assessment methods may have contributed to the wide range of the reported prevalence rates.

This study analyzed the distribution of various forms of picky eating. The results revealed that 15.5% of the young children in our study did not like to eat vegetables. Vegetables often have bitter or strong flavors after cooking, which may be a reason for why many children do not eat them [2]. In addition, picky eaters were significantly more likely to dislike eating vegetables, fruits, meat, and seafood than non-picky eaters were. This finding is consistent with those of other studies, indicating that a common pattern among picky eaters is a lower intake of vegetables and, to a lesser extent, fruits than that of non-picky eaters [36,37,38]. Understanding the distribution of picky eating behaviors can provide insights into young children’s eating patterns and nutritional status and reveal possible research directions for future studies. For example, a meta-analysis of studies on parent-targeted home-based interventions indicated that higher exposure to different tastes substantially improved vegetable intake among children [39].

In this research, coparenting was divided into two types, namely supportive and undermining coparenting, and we evaluated the relations between these two types and young children’s picky eating behaviors. The significant, negative direct effect of supportive coparenting on picky eating behaviors highlights the key role of supportive coparenting in shaping young children’s eating behaviors. Our study also revealed that undermining coparenting had a significant, positive direct effect on picky eating behaviors in young children. These findings emphasize the different roles of supportive and undermining coparenting in shaping young children’s eating behaviors. This result is consistent with those of previous studies showing that coparenting was particularly beneficial for young children’s diets when the parents had cohesive dietary choices and collaborated to foster healthy eating habits, which increased the likelihood of their children adopting and maintaining such habits [40]. Lower coparenting agreement and support were associated with increased parenting stress, specifically stress related to challenges in managing disruptive behaviors during meals [41]. These studies corroborate our finding of the role of supportive coparenting being associated with lower picky eating behaviors and emphasize the need for a holistic approach that considers coparenting and parenting education programs, along with interventions, to promote healthy eating habits in children.

Our research is consistent with previous studies demonstrating the importance of positive parenting strategies in improving children’s diets [21,24]. For example, higher levels of maternal role modeling, encouragement with respect to having a diverse and balanced diet, and instruction regarding nutritional information were determined to be associated with increased vegetable and whole grain intake among children in a multiethnic Asian population [24]. Our findings indicate that the parents were most effective in encouraging and enabling their children to try new foods, with these activities scoring significantly higher than the others. This result indicates that parenting education should prioritize guiding, modeling, and teaching fun and engaging methods for introducing children to new foods, indicating their potential effectiveness in fostering healthier eating habits [28,42]. Addressing these aspects can guide parents aimed at promoting healthy eating behaviors among young children, and it can provide a practical foundation for parenting education to ensure young children receive well-rounded nutrition and foster their overall growth and development.

Our study explored the complex interplay between dietary coparenting, parenting strategies, and picky eating behaviors, highlighting the significant diminishing role of supportive coparenting in being associated with lower picky eating behaviors among young children. Our analysis revealed a negative association of supportive coparenting with picky eating behaviors and identified two key parenting strategies—encouraging and facilitating the trying of new foods and guiding and modeling—as significant mediators. The indirect effects of these strategies explained 45.0% (supportive) and 40.0% (undermining) of the total effect on the association between dietary coparenting, parenting strategies, and picky eating behaviors in children. One study reported that supportive coparenting did not lead to controlling feeding practices. By contrast, undermining coparenting was linked to using food for emotional regulation, offering food as a reward, and implementing weight-related restrictions but not to pressured eating or health-based dietary restrictions [43]. In addition, our results revealed that undermining coparenting was associated with more frequent picky eating behaviors, with its influence mediated by effective parenting strategies, further emphasizing the complexity of parental influence on children’s dietary practices. Our study stands out for its simultaneous exploration of the relations among dietary coparenting, parenting strategies, and young children’s picky eating behaviors, offering novel insights into the nuanced interplay between parenting partnership, parenting, and children’s eating behaviors in the family context within a culture.

Cross-sectional designs in this study may limit causal inferences, so it is suggested that future studies could conduct longitudinal designs. We invited parents from 14 preschools across different districts, offered appropriate incentives, and ensured the participants remained anonymous. However, the recruitment approach may have caused self-selection bias. Our research highlights the need to address sample limitations and calls for future research to include a broader range of participants, encompassing paired fathers and mothers, other caregivers (e.g., grandparents), and also single-parent families, to understand the full spectrum of dietary coparenting and parenting strategies. Investigating the interactions among various caregivers and the effects of supportive coparenting, as well as effective parenting strategies, offers deeper insights into children’s picky eating behaviors. Moreover, exploring the beliefs and motivations underlying parents’ choice of strategies and the impact of the strategies on children’s eating behaviors across potential genetic predispositions and cultural and socioeconomic backgrounds would enhance our understanding of the nurture and nature factors influencing picky eating. Furthermore, exploring more strategies for dealing with pickiness may help in developing a well-established scale. Such a comprehensive approach would not only improve the generalizability of findings but also contribute to the development of tailored guidance and educational programs that foster positive dietary coparenting, parenting strategies, and picky eating behaviors in children.

## 5. Conclusions

This study advances the understanding of managing picky eating in young children by highlighting the critical roles of dietary coparenting and targeted parental strategies. Our findings reveal that effective coparenting involves more than routine caregiving; it also involves strategic efforts that improve children’s nutritional intake and development, indicating a need for cohesive and collaborative parenting to foster healthier eating behaviors in children. Establishing healthy eating patterns early and establishing supportive eating environments are essential for preventing picky eating and ensuring long-term health benefits. The insights provided by this study can assist professionals, caregivers, and researchers in developing more effective strategies for promoting holistic development in children, and future researchers should explore the applicability of our findings across individuals of diverse cultural backgrounds and age groups.

## Figures and Tables

**Table 1:** Participants’ characteristics.

Variables	*n* (%)
Characteristics of parents	
Gender	
Mother	348 (85.3)
Father	60 (14.7)
Parent’s age (year)	
Mother	37.49 ± 4.46 ^a^
Father	39.66 ± 4.94 ^a^
Education level of the mother	
≤High school	69 (16.9)
University	273 (66.9)
Graduate school	66 (16.2)
Education level of the father	
≤High school	97 (23.8)
University	221 (54.2)
Graduate school	90 (22.1)
Characteristics of young children	
Gender	
Boy	229 (56.1)
Girl	179 (43.9)
Age (month)	64.52 ± 11.22 ^a^
≤47 months	42 (10.3)
48–60 months	179 (43.9)
≥61 months	270 (66.2)
Height (cm)	111.40 ± 7.94 ^a^
Weight (kg)	19.18 ± 3.69 ^a^
BMI	
Underweight	45 (11.0)
Normal weight	306 (75.0)
Overweight	28 (6.9)
Obesity	29 (7.1)

^a^ Data are presented as the mean ± standard deviation.

**Table 2:** Dietary coparenting scores.

Dietary Coparenting	Item	Mean Score ^a^ (SD)
Supportive		3.83 (0.73)
	My spouse is supportive of my approach to disciplining our child’s eating behavior.	3.84 (0.82)
	My spouse agrees with the guidance I provide regarding our child’s eating behavior.	3.78 (0.86)
	When I emphasize the importance of a balanced diet to our child, my spouse complies with the request.	3.91 (0.83)
	My spouse provides additional explanations to our child regarding my expectations for eating behavior.	3.67 (0.93)
	When I ask my spouse for help in disciplining our child’s eating behavior, my spouse is willing to assist.	3.95 (0.80)
Undermining		1.82 (0.72)
	My spouse criticizes my approach to disciplining our child’s eating behavior in front of the child.	1.90 (0.84)
	My spouse engages in arguments with me regarding how to discipline our child’s eating behavior.	1.87 (0.83)
	My spouse interferes with my efforts to discipline our child’s eating behavior.	1.82 (0.80)
	When I handle our child’s eating behavior, my spouse undermines me.	1.72 (0.77)
	My spouse opposes the rules I set for our child’s eating behavior.	1.78 (0.77)

^a^ Five-point Likert scale (1–5, strongly disagree–strongly agree).

**Table 3:** Parenting strategy scores.

	Item	Mean Score ^a^ (SD)
Encouraging and facilitating the trying of new foods		3.88 (0.57)
	After my child tries a new food, I provide positive encouragement and affirmation.	4.23 (0.73)
	If my child encounters an unfamiliar or undesirable food, I encourage them to start with a small portion.	4.21 (0.76)
	When faced with an unfamiliar or undesirable food, I explain the benefits of the food to encourage my child to eat it.	4.13 (0.80)
	I introduce new foods for my child to try.	3.61 (0.72)
	I present different types of food attractively on the plate to encourage my child to try them.	3.23 (1.07)
Co-preparing meals and providing meal variations		3.00 (0.61)
	I use multiple cooking methods for my child, including boiling, stir-frying, and pan-frying.	3.81 (0.84)
	I incorporate foods in different ways, such as chopping cauliflower, bell peppers, or carrots into small pieces, to increase opportunities for my child to try them.	3.30 (1.03)
	I go grocery shopping with my child to enhance their interest in food.	2.93 (0.92)
	I involve my child in the food preparation process to foster their interest in food.	2.71 (0.83)
	I work with my child to make food into cute patterns or shapes.	2.28 (0.90)
Guiding and modeling		3.38 (0.69)
	I facilitate joint dining experiences for my child to dine with their siblings or friends, thereby establishing positive learning opportunities.	3.85 (1.03)
	I model for my child by trying different foods myself.	3.77 (0.95)
	I cultivate enjoyable mealtime experiences to make dining feel like something to look forward to.	3.71 (0.94)
	I use picture books or videos to explain the importance of a balanced diet to my child.	2.89 (1.07)
	I play games to help my child become familiar with various foods.	2.69 (1.04)

^a^ Five-point Likert scale (1–5, never–always).

**Table 4:** Young children’s picky eating behavior scores.

	Item	Mean Score ^a^ (SD)
Picky eating behaviors		2.48 (0.73)
	My child is picky and eats only or refuses to eat specific types of food (excluding religious or allergy factors).	2.93 (0.96)
	My child refuses to eat new foods (i.e., those not tried before).	2.72 (0.89)
	My child enjoys trying different types of food. (reverse scoring)	2.52 (0.98)
	My child eats only foods prepared in a particular way.	1.76 (0.81)

^a^ Five-point Likert scale (1–5, never–always).

**Table 5:** Comparison of non-picky and picky eating group for young children’s preferences for different types of food.

	Non-Picky Eating Group		Picky Eating Group		
	Mean ^a^	SD	Mean ^a^	SD	*t* ^b^
Total average	1.63	0.55	2.42	0.69	−11.52 ***
My child does not like to eat vegetables.	1.90	0.91	3.16	1.03	−12.36 ***
My child does not like to eat fruits.	1.30	0.57	1.92	1.01	−6.52 ***
My child does not like to eat meat (excluding religious reasons).	1.71	0.82	2.27	1.07	−5.24 ***
My child does not like to eat seafood (excluding allergy reasons).	1.66	0.81	2.47	1.13	−7.23 ***
My child eats only rice or noodles.	1.57	0.87	2.31	1.07	−6.93 ***

^a^ Five-point Likert scale (1–5, never–always). ^b^ Independent sample *t* test. *** *p* < 0.001.

**Table 6:** Correlations among dietary coparenting, parenting strategies, and young children’s picky eating behaviors.

	01	02	03	04	05
01 Supportive	1				
02 Undermining	−0.48 ***	1			
03 Encouraging and facilitating the trying of new foods	0.41 ***	−0.17 ***	1		
04 Copreparing meals and providing meal variations	0.32 ***	−0.05	0.49 ***	1	
05 Guiding and modeling	0.37 ***	−0.15 **	0.56 ***	0.52 ***	1
06 Picky eating behaviors	−0.40 ***	0.20 ***	−0.46 ***	−0.35 ***	−0.40 ***

** *p* < 0.01. *** *p* < 0.001.

**Table 7:** Summary of mediation analysis.

Paths	Effect (95% CI)	Percentage %
Supportive → Picky eating behaviors		
Total effect	−0.40 (−0.49, −0.31)	100
Direct effect	−0.22 (−0.32, −0.13)	55
Indirect effect (Supportive → Parenting strategies → Picky eating behaviors)	−0.18 (−0.23, −0.12)	45
Undermining → Picky eating behaviors		
Total effect	0.20 (0.10, 0.30)	100
Direct effect	0.12 (0.03, 0.21)	60
Indirect effect (Undermining → Parenting strategies → Picky eating behaviors)	0.08 (0.03, 0.13)	40

## Data Availability

The data used in this study are unavailable because of privacy and ethical restrictions.

## References

[1] van den Brand A.J.P., Hendriks-Hartensveld A.E.M., Havermans R.C., Nederkoorn C. (2023). Child characteristic correlates of food rejection in preschool children: A narrative review. Appetite.

[2] Chang Y., Liu M., Lo F., Wang K. (2018). Early Childhood Nutrition Reference Manual.

[3] Finistrella V., Manco M., Ferrara A., Rustico C., Presaghi F., Morino G. (2012). Cross-sectional exploration of maternal reports of food neophobia and pickiness in preschooler-mother dyads. J. Am. Coll. Nutr..

[4] Sandvik P., Ek A., Eli K., Somaraki M., Bottai M., Nowicka P. (2019). Picky eating in an obesity intervention for preschool-aged children—What role does it play, and does the measurement instrument matter?. Int. J. Behav. Nutr. Phys. Act..

[5] Tharner A., Jansen P.W., Kiefte-de Jong J.C., Moll H.A., van der Ende J., Jaddoe V.W., Hofman A., Tiemeier H., Franco O.H. (2014). Toward an operative diagnosis of fussy/picky eating: A latent profile approach in a population-based cohort. Int. J. Behav. Nutr. Phys. Act..

[6] Taylor C.M., Emmett P.M. (2019). Picky eating in children: Causes and consequences. Proc. Nutr. Soc..

[7] Taylor C.M., Wernimont S.M., Northstone K., Emmett P.M. (2015). Picky/fussy eating in children: Review of definitions, assessment, prevalence and dietary intakes. Appetite.

[8] da Costa M.P., Severo M., Oliveira A., Lopes C., Hetherington M., Vilela S. (2022). Longitudinal bidirectional relationship between children’s appetite and diet quality: A prospective cohort study. Appetite.

[9] Jansen P.W., Roza S.J., Jaddoe V.W., Mackenbach J.D., Raat H., Hofman A., Verhulst F.C., Tiemeier H. (2012). Children’s eating behavior, feeding practices of parents and weight problems in early childhood: Results from the population-based Generation R Study. Int. J. Behav. Nutr. Phys. Act..

[10] Kwok F.Y., Ho Y.Y., Chow C.M., So C.Y., Leung T.F. (2013). Assessment of nutrient intakes of picky-eating Chinese preschoolers using a modified food frequency questionnaire. World J. Pediatr..

[11] Marchi M., Cohen P. (1990). Early childhood eating behaviors and adolescent eating disorders. J. Am. Acad. Child Adolesc. Psychiatry.

[12] Taylor C.M., Northstone K., Wernimont S.M., Emmett P.M. (2016). Picky eating in preschool children: Associations with dietary fibre intakes and stool hardness. Appetite.

[13] Mascola A.J., Bryson S.W., Agras W.S. (2010). Picky eating during childhood: A longitudinal study to age 11 years. Eat. Behav..

[14] Pesch M.H., Bauer K.W., Christoph M.J., Larson N., Neumark-Sztainer D. (2020). Young adult nutrition and weight correlates of picky eating during childhood. Public Health Nutr..

[15] Taylor C.M., Hays N.P., Emmett P.M. (2019). Diet at age 10 and 13 years in children identified as picky eaters at age 3 years and in children who are persistent picky eaters in a longitudinal birth cohort study. Nutrients.

[16] Cano S.C., Hoek H.W., van Hoeken D., de Barse L.M., Jaddoe V.W., Verhulst F.C., Tiemeier H. (2016). Behavioral outcomes of picky eating in childhood: A prospective study in the general population. J. Child Psychol. Psychiatry.

[17] Dubois L., Bédard B., Goulet D., Prud’homme D., Tremblay R.E., Boivin M. (2022). Eating behaviors, dietary patterns and weight status in emerging adulthood and longitudinal associations with eating behaviors in early childhood. Int. J. Behav. Nutr. Phys. Act..

[18] Rubio B., Rigal N. (2017). Parental concerns and attributions of food pickiness and its consequences for the parent-child relationship: A qualitative analysis. J. Child Health Care.

[19] Goh D.Y., Jacob A. (2012). Perception of picky eating among children in Singapore and its impact on caregivers: A questionnaire survey. Asia Pac. Fam. Med..

[20] Balantekin K.N., Anzman-Frasca S., Francis L.A., Ventura A.K., Fisher J.O., Johnson S.L. (2020). Positive parenting approaches and their association with child eating and weight: A narrative review from infancy to adolescence. Pediatr. Obes..

[21] Scaglioni S., De Cosmi V., Ciappolino V., Parazzini F., Brambilla P., Agostoni C. (2018). Factors Influencing Children’s Eating Behaviours. Nutrients.

[22] Tan C.C., Domoff S.E., Pesch M.H., Lumeng J.C., Miller A.L. (2020). Coparenting in the feeding context: Perspectives of fathers and mothers of preschoolers. Eat. Weight Disord..

[23] Khandpur N., Charles J., Davison K.K. (2016). Fathers’ Perspectives on Coparenting in the Context of Child Feeding. Child Obes..

[24] Quah P.L., Syuhada G., Fries L.R., Chan M.J., Lim H.X., Toh J.Y., Sugianto R., Aris I.M., Lee Y.S., Yap F. (2018). Maternal feeding practices in relation to dietary intakes and BMI in 5 year-olds in a multi-ethnic Asian population. PLoS ONE.

[25] Douglas S., Darlington G., Beaton J., Davison K., Haines J., on behalf of the Guelph Family Health Study (2021). Associations between coparenting quality and food parenting practices among mothers and fathers in the Guelph Family Health Study. Nutrients.

[26] World Health Organization Body Mass Index—BMI. https://www.euro.who.int/en/health-topics/disease-prevention/nutrition/a-healthy-lifestyle/body-mass-index-bmi.

[27] Chen F., Li T. (2004). Parenting experiences of couples with young children: Division of labor and coparenting. Formosa J. Ment. Health.

[28] Podlesak A.K., Mozer M.E., Smith-Simpson S., Lee S.Y., Donovan S.M. (2017). Associations between parenting style and parent and toddler mealtime behaviors. Curr. Dev. Nutr..

[29] Wardle J., Guthrie C.A., Sanderson S., Rapoport L. (2001). Development of the children’s eating behaviour questionnaire. J. Child Psychol. Psychiatry.

[30] Hayes A.F. (2022). Introduction to Mediation, Moderation, and Conditional Process Analysis: A Regression-Based Approach.

[31] Sandvik P., Ek A., Somaraki M., Hammar U., Eli K., Nowicka P. (2018). Picky eating in Swedish preschoolers of different weight status: Application of two new screening cut-offs. Int. J. Behav. Nutr. Phys. Act..

[32] Steinsbekk S., Sveen T.H., Fildes A., Llewellyn C., Wichstrøm L. (2017). Screening for pickiness—A validation study. Int. J. Behav. Nutr. Phys. Act..

[33] Food and Drug Administration (2003). Early Childhood Nutrition Reference Manual.

[34] Haszard J.J., Skidmore P.M., Williams S.M., Taylor R.W. (2015). Associations between parental feeding practices, problem food behaviours and dietary intake in New Zealand overweight children aged 4–8 years. Public Health Nutr..

[35] Chao H.C. (2018). Association of picky eating with growth, nutritional status, development, physical activity, and health in preschool children. Front. Pediatr..

[36] Dubois L., Farmer A.P., Girard M., Peterson K. (2007). Preschool children’s eating behaviours are related to dietary adequacy and body weight. Eur. J. Clin. Nutr..

[37] van der Horst K., Deming D.M., Lesniauskas R., Carr B.T., Reidy K.C. (2016). Picky eating: Associations with child eating characteristics and food intake. Appetite.

[38] Xue Y., Lee E., Ning K., Zheng Y., Ma D., Gao H., Yang B., Bai Y., Wang P., Zhang Y. (2015). Prevalence of picky eating behaviour in Chinese school-age children and associations with anthropometric parameters and intelligence quotient. A cross-sectional study. Appetite.

[39] Touyz L.M., Wakefield C.E., Grech A.M., Quinn V.F., Costa D.S.J., Zhang F.F., Cohn R.J., Sajeev M., Cohen J. (2018). Parent-targeted home-based interventions for increasing fruit and vegetable intake in children: A systematic review and meta-analysis. Nutr. Rev..

[40] Mahmood L., Flores-Barrantes P., Moreno L.A., Manios Y., Gonzalez-Gil E.M. (2021). The influence of parental dietary behaviors and practices on children’s eating habits. Nutrients.

[41] Thullen M., Bonsall A. (2017). Co-parenting quality, parenting stress, and feeding challenges in families with a child diagnosed with Autism Spectrum Disorder. J. Autism Dev. Disord..

[42] Robinson C., Mandleco B., Olsen S., Hart C., Touliatos J., Perlmutter B., Holden G. (2001). Parenting Styles and Dimensions Questionnaire [PSDQ). Handbook of Family Measurement Techniques: Instruments & Index.

[43] Tan C.C., Herzog N.K., Mhanna A. (2021). Associations between supportive and undermining coparenting and controlling feeding practices. Appetite.

